# Cervical cancer screening in low- and middle-income countries: A systematic review of economic evaluation studies

**DOI:** 10.1016/j.clinsp.2022.100080

**Published:** 2022-07-26

**Authors:** Carmen Phang Romero Casas, Rita de Cássia Ribeiro de Albuquerque, Rafaela Borge Loureiro, Angela Maria Gollner, Marina Gonçalves de Freitas, Graciela Paula do Nascimento Duque, Juliana Yukari Kodaira Viscondi

**Affiliations:** a Centro de Desenvolvimento Tecnológico em Saúde (CDTS), Fundação Oswaldo Cruz (FIOCRUZ), Rio de Janeiro, RJ, Brazil; b Núcleo de Avaliação de Tecnologias em Saúde (NATS), Instituto Nacional de Câncer (INCA), Rio de Janeiro, RJ, Brazil; c Laboratório de Epidemiologia (Lab-Epi), Universidade Federal do Espírito Santo (UFES), Vitória, ES, Brazil; d Hospital Universitário da Universidade Federal de Juiz de Fora (HU-UFJF/ EBSERH), Juiz de Fora, MG, Brazil; e Câmara de Regulação do Mercado de Medicamentos (CMED), Agência Nacional de Vigilância Sanitária (ANVISA), Brazil; f Instituto Butantã, Hospital Alemão Oswaldo Cruz (HAOC), São Paulo, SP, Brazil

**Keywords:** Economic evaluations, Cervical cancer, DNA tests for human papillomavirus

## Abstract

Economic assessments are relevant to support the decision to incorporate more cost-effective strategies to reduce Cervical Cancer (CC) mortality. This systematic review analyzes the economic evaluation studies of CC prevention strategies (HPV DNA-based tests and conventional cytology) in low- and middle-income countries. Medline, EMBASE, CRD, and LILACS were searched for economic evaluation studies that reported cost and effectiveness measures of HPV DNA-based tests for CC screening and conventional cytology in women, without age, language, or publication date restrictions. Selection and data extraction were carried out independently. For comparability of results, cost-effectiveness measures were converted to international dollars (2019). Report quality was assessed using the CHEERS checklist. The Dominance Matrix Ranking (DRM) was used to analyze and interpret the results. The review included 15 studies from 12 countries, with cost-effectiveness analyzes from the health system's perspective and a 3% discount rate. The strategies varied in age and frequency of screening. Most studies used the Markov analytical model, and the cost-benefit threshold was based on the per capita GDP of each country. The sensitivity analysis performed in most studies was deterministic. The completeness of the report was considered sufficient in most of the items evaluated by CHEERS. The Dominance Interpretation (DRM) varied; in 6 studies, the HPV test was dominant, 5 studies showed a weak dominance evaluating greater effectiveness of the HPV test at a higher cost, yet in 2 studies conventional cytology was dominant. Although the context-dependent nature of economic evaluations, this review points out the challenge of methodological standardization in the analytical models.

## Highlights

• Screening and early detection programs are a cornerstone of cervical cancer prevention.
• Human papillomavirus DNA-based testing reduced the incidence of CC below per capita GDP.
• Cost-effectiveness of HPV testing versus cytology in Low- and Middle-Income Countries.

## Introduction

Cervical Cancer (CC) is in seventh place in the world ranking and is the fourth most common type in females. In 2020, 604,127 new cases were estimated worldwide, with an age-standardized rate of 13.3 per 100,000 women and 341,831 deaths from this neoplasm.^1^

According to the World Health Organization (WHO) data, CC has become rare in high-income countries, but it is still the main cancer cause of mortality among women in low- and middle-income countries. Age-standardized and mortality rates are higher in South, East, and West Africa, and Melanesia, and lower in Western Europe, North America, Australia, New Zealand, and Western Asia.^2^

In November 2020, the Global Strategy for the Elimination of Cervical Cancer was launched during the World Health Assembly. If effective actions are not taken, the prevalence of the disease could increase to 700,000 cases by 2030, and the number of deaths could reach 400,000 each year in the next decade, according to the WHO.^1^

Vaccination, screening, and treatment are the cornerstones to implement the strategy, which had the adhesion of 194 countries. The proposed global targets are 90% coverage of Human Papillomavirus (HPV) vaccination in girls under 15, 70% coverage with HPV testing among women aged 35 to 45 years old, and 90% coverage of treatment, including palliative care.^1^

The main promoter of CC is the Human Papillomavirus (HPV), which mainly affects women over 30 years old, with a peak incidence in 45 to 50 years old. The disease develops slowly and has a long phase before becoming invasive. If diagnosed in the early stages, it is treatable and more likely to be cured through adequate screening, early detection, and treatment, which is cheaper for the health system.^3^

The Pap smear test (Papanicolaou Test) is one of the screening methods used as a strategy to detect CC. Its main favorable aspect is the low cost.^4^

In countries where this method is widely offered in organized public health programs, cervical cytology has significantly reduced incidence and mortality, particularly in countries with high target population coverage, control, and quality assurance associated with the program.^5^

One of the limitations of cytology-based screening is the low sensitivity for the detection of precursor lesions (cervical intraepithelial neoplasm [CIN2+] grade 2 or higher) compared to HPV testing.^6^ Another limitation is the complexity of the logistical and care infrastructure to implement quality control and carry out the appropriate clinical management of women with positive screening. For these reasons, cervical cytology screening has not yet reached high population coverage in low- and middle-income countries, where it usually occurs opportunistically.^7^^,^^8^

The discovery that persistent infections with a few genetically related HPV types cause virtually all cases of CC has led not only to vaccine development but also to HPV testing. HPV testing for (high risk) carcinogenic types of HPV infections is more sensitive than cytology, allowing for greater safety and longer screening intervals.^9^

The implementation of screening and early detection programs is one of the cornerstones of cancer prevention. Despite evidence that early detection saves lives, global disparities in access to services persist. Economic assessments are relevant to support the decision to incorporate and implement the most cost-effective strategies available to reduce female mortality from CC, especially in low- and middle-income countries.^10^

For low- and middle-income countries, which often face budgetary constraints in their health systems, achieving the goals proposed by the WHO requires investments in cost-effective interventions. Thus, it is necessary to consolidate evidence and optimize the distribution of resources in these locations.

This Systematic Review (SR) aimed to analyze the cost-effectiveness of CC screening strategies by comparing the molecular tests for HPV and the Pap smear test used in women from low- and middle-income countries.

## Methods

This SR was performed according to the guidelines of the Center for Reviews and Dissemination (CRD)^11^ and reported according to the Preferred Reporting Items for Systematic Reviews and Meta-Analyses checklist.^12^ The protocol was previously registered in PROSPERO (CRD42020208135).^13^

### Eligibility criteria

Economic evaluation studies that reported cost-related results, such as Incremental Cost-Effectiveness Ratio (ICER); Incremental Cost-utility Ratio (ICUR); cost difference; incremental costs, and measures of effectiveness such as Years of Life Lost (YLL); Years of Life Saved (YLS); Quality-Adjusted Life Years (QALY) and Disability-Adjusted Life Years (DALY) from HPV DNA-based testing for cervical cancer screening and conventional cytology, in women from low- and middle-income countries, without age, language or publication date restrictions.

Were excluded studies performed with populations from high-income countries, hysterectomized women, HIV+ and screening performed by visual inspection of the cervix, liquid cytology, or computer-assisted automated. Publications that were not economic studies (clinical studies, systematic reviews) or preliminary studies (conference abstracts) were excluded.

### Sources of information and searches

The search for economic evaluation studies was carried out in August 2020 in the MEDLINE databases via PubMed, EMBASE, CRD (Centre for Review and Dissemination), and Latin American and Caribbean Literature in Health Sciences (LILACS) via the Virtual Health Library (BVS). Also, a manual search was performed in the reference list of the selected publications. In addition to terms related to screening methods, search strategies included terms related to cost-effectiveness (human papillomavirus tests; HPV test; Papanicolaou test; pap smear; economic evaluation) and were made available in the supplementary material (Chart 1).

### Study selection

The selection was made by two researchers independently, who initially read titles and abstracts and then evaluated the full texts, using the Rayyan program^14^ and the Mendeley Reference Manager (version 1.19.8. Elsevier, London, UK)^15^ to exclude duplicates and merge records from different databases. Disagreements were discussed and solved by consensus or, eventually, by a third researcher.

### Data extraction

Double-blinded data extraction was performed using a form previously prepared in an Excel spreadsheet (Microsoft Corp., Redmond, WA). Information extracted from selected articles included study characteristics (author and year of publication, location, and economic classification according to the World Bank, type of economic study and modeling, time horizon, payer perspective, and discount rate), population characteristics, tracking strategies used and results of cost and effectiveness measures.

To enable comparisons between studies carried out in different years, countries, and considering other currencies and accounting for the effects of inflation, the ICER measures were converted to international dollars and updated for the year 2019. In this process, the authors used the FXTOP 2001‒2020 tool,^16^ the site for currency conversion and historical exchange rates, and the purchasing power parity tables provided by the World Bank.^17^ A descriptive synthesis including the analytic approach of the studies contextualized by geographical regions and countries’ incomes were presented in tables.

### Assessment of the report of economic evaluation studies

As there was no tool to assess the risk of bias in economic evaluations, the Consolidated Health Economic Evaluation Reporting Standards^18^ (CHEERS) checklist was used as an instrument to verify whether the studies included in the SR contained the information considered essential.

### Classification of studies according to dominance ranking

The Dominance Ranking Matrix (DRM) system, developed by the Joanna Briggs Institute,^19^ was used to summarize and interpret the results of the economic evaluations. DRM provides three classification options: strong domain for intervention, weak domain for intervention, and no dominance for intervention. This hierarchical dominance matrix allows a visual summary of various economic analyses with different outcome measures (e.g., cost-effectiveness, cost-utility, cost-benefit) that otherwise would not be possible in a quantitative meta-analysis approach.^20^^,^^21^ The authors emphasize that, although DRM is not a method of quantitative synthesis, its hierarchical structure allows for an interpretation of the levels of the dominance of an intervention based on the assessment of benefits for costs and health outcomes in a study.

## Results

**Fig. 1:**
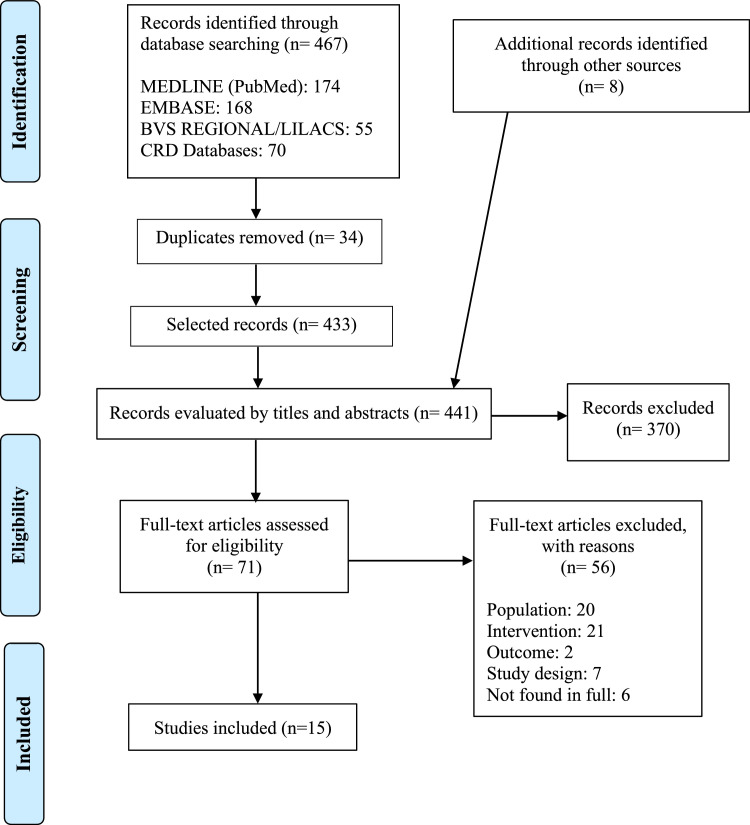
PRISMA flow diagram showing the identification, selection, eligibility, and inclusion of studies in the systematic review.

Four hundred sixty-seven potentially relevant publications were retrieved from the electronic databases, and 8 were identified through a manual search in the reference lists. After excluding duplicates (n = 34), 441 publications were chosen for the initial selection, of which 370 references were excluded. Seventy-one articles were selected for full reading, and of these, 15 were included in the SR (Fig. 1). The excluded studies, together with the reasons for their exclusions, were included in the supplementary material (Chart 2).

Table 1 presents the characteristics of the 15 studies included,^22^, ^23^, ^24^, ^25^, ^26^, ^27^, ^28^, ^29^, ^30^, ^31^, ^32^, ^33^, ^34^, ^35^, ^36^ published between 2002 and 2019, in 12 countries on three different continents: Africa, Asia, and America. According to the World Bank classification, most studies were conducted in upper-middle-income countries (71.4%).^37^

**Table 1:** Characteristics of the studies included in the systematic review.

Author, year	Country	Income classification (WB)	Study Type	Model	Perspective	Time horizon	Discount Rate	Population Age (years)	Index Test	Reference Test
Andrés-Gamboa (2008)	Colombia	Upper-middle	CEA	Markov decision model	Payer	Throughout life	3%	21‒69 (cytology)	HPV DNA	Cervical smear
								30‒69 (HPV DNA)		Pap smear
Beal (2014)	Mexico	Upper-middle	CEA, CA	Decision Tree Model	Healthcare system	3 to 5 years	5%	35‒64	HPV DNA	Cervical smear
										Pap smear
Caetano (2006)	Brazil	Upper-middle	CEA	Decision Tree Model	Healthcare system	1 year	Without Information	25‒59	HPV DNA	Cervical smear
										Pap smear
Campos (2015)	India, Nicaragua and Uganda	Lower-middle India and Nicaragua	ACE, BIA	Microsimulation model	Health system and patient	Throughout life	3%	25‒50	HPV DNA	Cervical smear
		Low: Uganda								Pap smear
Campos (2017)	Nicaragua	Medium-Low	CEA	Microsimulation model	Health system and patient	Throughout life	3%	30‒59	HPV DNA	Cervical smear
										Pap smear
Flores (2011)	Mexico	Upper-middle	CEA	Decision Tree Model	Healthcare system	1 year	3%	20‒80	HPV DNA	Cervical smear
										Pap smear
Goldie (2005)	South Africa, India, Peru, Kenya and Thailand	Upper-middle: South Africa, Peru and Thail and Lower-middle: India and Kenya	CEA	Markov decision model	Health system and patient	Throughout life	3%	1 × in life: 35	Hybrid Capture II HPV test	Cervical smear
								2 × in life: 35 and 40		Pap smear
								3 × in life: 35, 40 and 45		
Gutierrez-Delgado (2008)	Mexico	Upper-middle	CEA	Markov decision model	Payer	20 years/intervention funding	3%	25‒64 (cytology)	Hybrid Capture II HPV test	Cervical smear
						100 years/health benefits		30‒69 (HPV DNA)		Pap smear
Levin (2010)	China	Upper-middle	CEA	Markov decision model	Health system and patient	Throughout life	3%	1 × in life: 35	Hybrid Capture II HPV test and HPV DNA	Cervical smear
								2 × in life: 35 and 40		Pap smear
								3 × in life: 35, 40 and 45		
Mandelblatt (2000).	Thailand	Upper-middle	CBA	Simulation model (Semi-Markov)	Health system and patient	Throughout life	3%	35‒55	Hybrid Capture II HPV test	Cervical smear
										Pap smear
Nahvijou (2014)	Iran	Upper-middle	CMA	Decision Tree Model	Healthcare system	Without Information	Without Information	35 years	HPV DNA	Cervical smear
										Pap smear
Nahvijou (2016)	Iran	Upper-middle	CEA	Markov decision model	Healthcare system	Throughout life	3%	21‒65	HPV DNA	Cervical smear
										Pap smear
Tantitamit (2015)	Thailand	Upper-middle	CEA	Decision Tree Model	Health system and patient	Without Information	Without Information	Without Information	Hybrid Capture II HPV test	Cervical smear
										Pap smear
Termrungruanglert (2017)	Thailand	Upper-middle	CEA	Markov decision model	Healthcare system	35 years	3%	35‒65	HPV DNA with 16/18 genotyping	Cervical smear
										Pap smear
Termrungruanglert (2019)	Thailand	Upper-middle	ACE, BIA	Markov decision model	Healthcare system	10 years	3.5%	35‒65	HPV DNA with 16/18 genotyping	Cervical smear
										Pap smear

CEA, Cost-Effectiveness Analysis; CA, Cost Analysis; CBA, Cost-Benefit Analysis; CMA, Cost-Minimization Analysis; BIA, Budget Impact Analysis; WB, World Bank.

Thirteen Cost-Effectiveness Analyses (CEA), one cost-minimization, and one cost-benefit analysis were found. The CEAs were complemented with budget impact analyses in 2 studies (Table 1). The analyses considered the health system perspective (n = 7),^23^^,^^24^^,^^27^^,^^32^^,^^33^^,^^35^^,^^36^ followed by the patient and health system perspective (n = 6) and ^25^^,^^26^^,^^28^^,^^30^^,^^31^^,^^34^ the payer perspective (n = 2).^22^^,^^29^

The predominant analytical models were the Markov (n = 7) ^22^^,^^28^^,^^29^^,^^30^^,^^33^^,^^35^^,^^36^ and Decision Tree models (n = 5).^23^^,^^24^^,^^27^^,^^32^^,^^34^ Microsimulation models (n = 2) ^25^^,^^26^ and the Semi-Markov model (n = 1) were also used.^31^

Table 2 summarizes the main results of the economic analysis studies. Cost-effectiveness values varied between studies due to the different strategies evaluated regarding age (35, 40, 45 years) and frequency of screening (1, 2, 3 times throughout life).

**Table 2:** Results of the studies included in the systematic review.

Author (Year)	Compared tracking strategies	Effective Measures	Types of costs	Currency (year)	Cost-effectiveness combined result	Exchange into international dollar (2019)	Cost-effectiveness threshold	Sensitivity analysis	Parameters Analyzed
Andrés-Gamboa	No tracking/ Conventional cytology/ DNA-HPV test	Years of life saved	Direct medical costs	US Dollar (2006)	USD44/ YSL	Int$77.42	USD 3,200	Without Information	Performance and costs of screening tests, diagnosis, and treatment costs for HSIL
Beal (2014)	Conventional cytology compared to: HR-HPV plus Conventional cytology; HR-HPV plus molecular screening; Co-testing	Number of missed cases of CIN 2, CIN 3 or cervical cancer avoided	Direct medical and non-medical costs	USD (2013)	ICER: HR-HPV plus Conventional Cytology USD 108.99; HR-HPV plus molecular screening USD 819; Co-testing USD-537	HR-HPV plus Conventional Cytology: Int$197.23 HR-HPV plus Molecular Screening: Int$1,482.03 Co-testing: Int $971.74	Willingness-to-pay 0 to 3,000 USD	Probability/ Monte Carlo Simulation	‒
Caetano (2006)	Conventional cytology/ Cytology in liquid medium/ CH-HPV test/ CH-HPV with self collection/ Conventional cytology with CH-HPV/ Cytology in liquid medium with CH-HPV	Number of detected cases of precursor lesions with a high degree of malignancy or cervical cancer	Direct medical costs	Brazilian Real BRL (2005)	BRL 1,404.36/ detected case of cancer or high-grade precursor lesion	Int$1,309.68	Without Information	Univariate Deterministics	Costs of Compared Strategies
Campos (2015)	HPV-DNA testing (provider-collected [cervical] and self-collected [vaginal] sampling), Visual inspection with acetic acid (VIA)/ Conventional cytology Screening strategies included several scenarios, varying both age at onset and frequency throughout life: 1x in life (25, 30, 35, 40, 45 or 50 years); 2x in life (25 and 35; 30 and 40 or 35 and 45 years); 3x in life (25, 35 and 45 years; 30, 35 and 40 years; 35, 40 and 45 years; or 30, 40 and 50 years)	Years of life saved	Direct medical costs	International Dollar Int $ (2011)	DNA-HPV test (cervical sampling) India: 1 × ICER in life (40 years: Int$330/YLS; 45 years: Int$190/YLS); 2 × in life (35 and 45 years: Int$390/YLS); 3 × in life (30, 35 and 40 years: Int$1,600/YLS; 30, 40 and 50 years: Int$580/YLS)	India: 1 × in life (40 years Int$402.53; 45 years Int$231.76); 2 × in life (35 and 45 years Int$475.72); 3 × in life (30, 35 and 40 years Int$1,951.68; 30, 40 and 50 years Int$707.49)	Int$ 5,240 GDP per capita in India	Probability/ Monte Carlo Simulation	Prevalence of age-specific high-risk HPV and age-specific incidence of cancer
					Nicaragua: ICER 2 × in life (30 and 40 years: Int$50/YLS); 3 × in life (30, 35 and 40 years: Int$ 180/YLS, 25, 35 and 45 years: Int$ 1200/YLS)	Nicaragua: 2 × in life (30 and 40 years Int$58.27); 3 × in life (30, 35 and 40 years Int$209.77; 25, 35 and 45 years Int$1,398.45)	Int$ 4,220 GDP per capita in Nicaragua		
					Uganda: ICER 1 × in life (35 years: Int$ 160/YLS; 40 years: I $120/YLS); 2 × in life (30 and 40 years: Int$210/YLS); 3 × in life (30, 40 and 50 years: Int$350/YLS)	Uganda: 1 × in life (35 years Int$166.32; 40 years Int$124.74); 2 × in life (30 and 40 years Int$ 218.29); 3 × in life (30, 40 and 50 years Int$ 2,165.77)	Int$1,370 GDP per capita in Uganda		
Campos (2017)	Conventional cytology w/3 years, with referral for colposcopy if ASCUS or worse result/ DNA-HPV test w/5 years, with referral for cryotherapy for HPV-positive eligible/ DNA-HPV test w/5 years, with referral for screening with visual inspection with acetic acid (VIA) for HPV-positive/ DNA-HPV test w/5 years, with referral to conventional cytology for HPV-positive	Years of life saved	Direct medical and non-medical costs	US Dollar U$ (2015)	USD320/ YLS	Int$ 969.36	USD 2,090 GDP per capita	Univariate Deterministics	Test performance, colposcopy performance, screening coverage, visit compliance, eligibility for cryotherapy after a positive screening and screening test, treatment effectiveness, discount rate, and costs
Flores (2011)	No screening/ Conventional cytology/ Self-collection DNA-HPV test/ DNA-HPV test administered by the doctor/ DNA-HPV test administered by the doctor plus Conventional cytology	Number of detected cases of high grade, cervical intraepithelial neoplasm or cervical cancer	Direct medical costs	US Dollar U$ (2008)	USD 9,352.00/ case detected	Int$ 21,581.07	Without Information	Univariate Deterministics	Performance and costs of screening tests and treatment costs
Goldie (2005)	Strategies vary according to the number of clinic visits, frequency of screening, and specific ages.	Years of life saved	Direct costs (medical and non-medical) and programme costs	International Dollar Int$ (2000)	Lifetime tracking (1, 2, 3 times, respectively)	India: 3 × in life Int$37.58, Kenya: 2 × in life Int$197.22; 3 × in life Int$ 310.24 Peru: 1 × in life Int$ 5.47; 2 × in life Int$ 16.31; 3 × in life Int$41.24 South Africa: 1 × in life Int$5.30; 2x in life Int$ 12.40; 3 × in life Int$ 27.89 Thailand: 1 × in life Int$ 3.44; 2 × in life Int$6.26; 3 × in life Int$ 13.29	Int$2,330 GDP per capita in IndiaI	Univariate Deterministics	Costs associated with invasive cancer treatment and target age of screening, while the choice between strategies was sensitive to test characteristics and screening costs.
					Costs/ YLS		Int$1,005 GDP per capita in Kenya		
					India = D; D; 24.32				
					Kenya = D; 70.15; 110.35		Int$4,747 GDP per capita in Peru		
					Turkey = 3.2; 9.54; 24.12				
					South Africa = 4.92; 11.52; 25.91		Int$9,486 GDP per capita in South Africa		
					Thailand = 2.67; 4.86; 10.32				
							Int$6,373 GDP per capita in Thailand		
Gutiérrez-Delgado (2008)	Strategies include 10 scenarios: 3 with screening by conventional Cytology, DNA-HPV Test (CH) or combined; and 7 with HPV vaccine	Years of life saved	Direct medical costs	Mexican Peso Mex$ (2006)	Conventional cytology by the Program, 80% coverage = ICER: Mex$16,678/YLS	Conventional cytology by the Program, 80% coverage: Int$3,078.93	Mex$ 88,688 GDP per capita	Univariate Deterministics	The frequency and cost of tracking for the HC-HPV test and the discount rate
					DNA-HPV w/3 years, 80% coverage = ICER: Mex$ 21,914/ YLS	DNA-HPV with 3 years, 80% coverage: Int$ 4,045.55			
Levin (2010)	Conventional cytology/ DNA-HPV test (CH-HPV, Rapid HPV test)	Years of life saved, Number of cancer cases avoided	Direct costs (medical and non-medical) and programme costs	US Dollar U$ (2005)	$50/ YLS for a single lifetime screening using county-level HPV DNA rapid testing compared to no screening.	DNA-HPV rapid test 1 × in life: Int$ 140.65 2 × in life: Int$ 225.04 3 × in life: 421.95	USD 1,702GDP per capita	Univariate Deterministics	Costs associated with invasive cancer, treatment of precancerous lesions, and screening test costs.
	The strategies varied according to age, screening frequency, number of clinic visits (1, 2 or 3) and service delivery configuration (city, county or national)				$80 and $150/ YLS two or three times in a lifetime, with the same strategy, respectively				
Mandelblatt (2002)	Strategies include 7 scenarios: 3 with screening per test: Conventional Cytology, VIA Test??, DNA-HPV Test and Combined Tests	Years of life saved	Direct medical and non-medical costs	US Dollar U$ (2000)	Incremental ratio ($/YL)	Variation from Int$ 621.47 to Int$ 34,514.67	Without Information	Probability/ Monte Carlo Simulation	Individual parameters: sensitivity and test cost, prevalence rates; and parameter combinations: two- or three-way sensitivity analysis at reasonable intervals to examine the robustness of model results
					All strategies saved lives, at costs ranging from $121 to $6,720/ YLS				
Nahvijou (2014)	Conventional cytology/ DNA-HPV test with conventional cytology	Not applicable^a^	Direct medical costs	International Dollar Int$ (2010)	Total cost/woman: Conventional cytology = $36.1; DNA-HPV Test USD 174.0	Conventional cytology: Int$ 18.84 DNA-HPV Test: Int$ 90.81	per capita GDP of the country	Without Information	Without Information
Nahvijou (2016)	11 screening strategies compared to no screening, varying both age at onset for Conventional cytology (21, 30, 35 years), for DNA-HPV Test (30, 35 years) and the interval between tests for Conventional cytology (3, 5, 10 years) for DNA-HPV Test (5, 10 years)	Life Years Saved, Quality Adjusted Life Years (QALY)	Direct medical costs	International Dollar Int$ (2013)	ICER $8,875/QALY compared to no tracking	Int$ 23,281.74	USD 6,631 GDP per capita	Univariate Deterministics	Performance and Costs of Tracking Tests
Tantitamit (2015)	Women Population with ASCUS results: Repeat conventional cytology/ Screening with DNA-HPV test/ Immediate colposcopy	Number of CIN 2+ cases detected	Direct medical costs	Thai Baht ₿ (2013)	Health system: ICER = 56,048 THB/ additional case of CIN 2+ detected	Health system: Int$ 4.62	USD 6,168.30 GDP per capita	Univariate Deterministics	Tracking Strategies Costs
					Patient: ICER = 62,712 THB/ additional case of CIN 2+ detected	Patient: Int$ 5.17			
Termrungruanglert (2017)	DNA-HPV test with 16/18 genotyping, with referral for colposcopy if positive or return to routine screening within 5 years/ HR-HPV test w/5 years, colposcopy for women with positive result/ Conventional cytology, followed by colposcopy if the result is ASCUS or worse	Number of detected cases of CIN 2, CIN 3 and cervical cancer per 100,000 women	Direct medical costs	Thai Baht ₿ (2016)	ICER test DNA-HPV with 16/18 genotyping: −360.810 THB (dominated)	DNA-HPV test with 16/18 genotyping: Int$-29.51HR-HPV test: Int$ 3,358.97	USD 5,904.20 GDP per capita	Univariate Deterministics	Prevalence of HPV infection in strategies 1 and 2, sensitivity of conventional cytology in strategy 3, discount rate and costs of all screening tools
					HR-HPV test: 41,075.1 THB/ Case detected	Conventional cytology: Int$ -941.39			
					Conventional cytology: −11,511.8 THB (dominated)				
Termrungruanglert (2019)	DNA-HPV test/double staining with 16/18 genotyping/ Conventional cytology	Number of pre-invasive and invasive cervical cancer cases identified quality-adjusted life years (QALY)	Direct medical costs	US Dollar U$ (2018)	ICER = USD1,395/QALY earned	Int$ 3,672.46	USD5,901 GDP per capita	Univariate Deterministics	Tracking Strategies Costs

ASCUS, Atypical Squamous Cells of Undetermined Meaning; HSIL, High-grade squamous intraepithelial lesion; HR-HPV, High-risk HPV test; CH-HPV, Hybrid Capture for HPV; YLS, Years of life saved; CER, cost-effectiveness ratio; ICER, Incremental cost-effectiveness ratio; D, Dominant.

a This is a cost-minimization study.

The outcome measures used were Years of Life Saved (YLS), Quality-Adjusted Life Years (QALY), and detected cases of high malignancy precursor lesions or cervical cancer.

Cost measures based on the perspective adopted included: costs per woman screened/100,000, lifetime costs, direct costs (medical and non-medical), and programmatic costs. Only 4 studies showed results in international dollars,^25^^,^^28^^,^^32^^,^^33^ the others used US dollars ^22^^,^^23^^,^^26^^,^^27^^,^^30^^,^^31^^,^^36^ or local currencies.^24^^,^^29^^,^^34^^,^^35^

Regarding the cost-effectiveness threshold, most studies (n = 10) used the method recommended by the Commission on Macroeconomics and Health of the World Health Organization,^37^ which establishes the value of the Gross Domestic Product (GDP) *per capita* of the country as a parameter to determine whether a technology is considered cost-effective (Table 2).

In 7 studies, the authors chose to present the cost-effectiveness threshold in US dollars.^22^^,^^26^^,^^30^^,^^33^, ^34^, ^35^, ^36^ Only 2 studies presented values in international dollars.^25^^,^^27^ In one publication, the value was presented in local currency; ^29^ in another, *willingness to pay* was used as a measure,^23^ and 3 studies provided no information.^24^^,^^27^^,^^31^

As the *per capita* income of the included countries is relatively low (low and middle-income countries, with GDP between USD 1,702 and USD 6,631 or Int$ 1,005 and Int$ 9,486), the HPV DNA test was considered cost-effective in different scenarios.

The sensitivity analysis performed in most studies was deterministic (n = 10),^24^^,^^26^, ^27^, ^28^, ^29^, ^30^^,^^33^, ^34^, ^35^, ^36^ only 3 studies presented a probabilistic analysis ^23^^,^^25^^,^^31^ and 2 authors did not report it.^22^^,^^32^ Among the parameters under analysis, those that significantly impacted the results were identified as sensitivity and specificity of competing tests; costs of HPV DNA testing and Pap smears; the prevalence of age-specific HPV, and the incidence rates of cervical cancer.

**Fig. 2:**
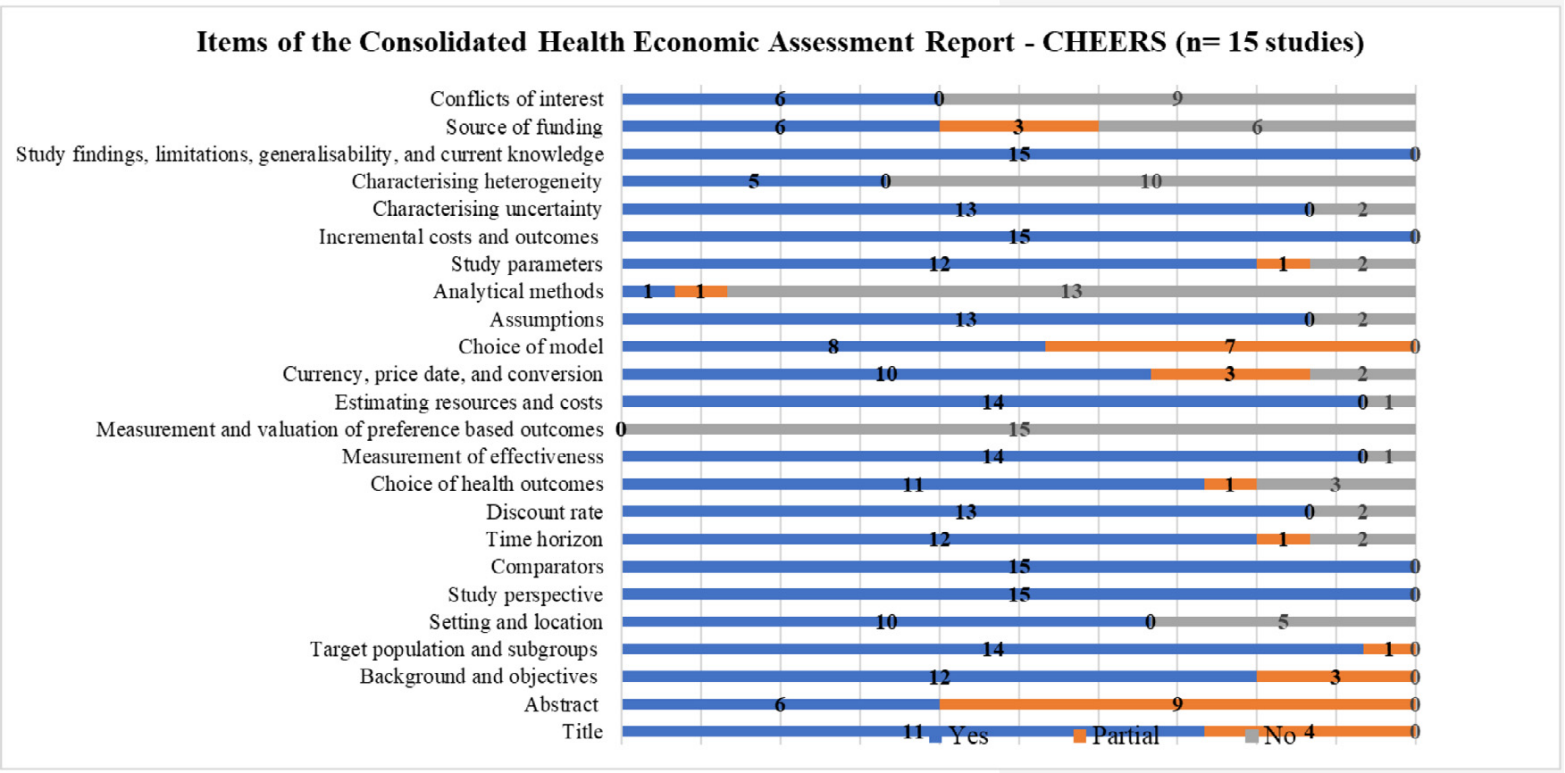
Items reported in the included studies, according to CHEERS.

Fig. 2 summarizes the items reported in the studies included in the SR, according to CHEERS.^18^ It is important to highlight that this *checklist* is used to prepare economic evaluation studies and not analyze the methodological quality itself. It was used to examine the 24 items that ideally should be included in publications on economic evaluation in health.

For all economic evaluations, data on study perspectives, comparators, costs and outcomes, findings, and limitations were included, however, data on analytical methods was the least reported item in the studies.

Six of the studies included (40%)^22^^,^^28^^,^^30^^,^^33^^,^^35^^,^^36^ presented transition probabilities; only one study explained the calculation of these probabilities or whether cycle correction was used, which are fundamental aspects in the development of Markov models. Only one study^25^ (6.66%) reported the use of some calibration method.

None of the studies presented justification for the duration of the cycle, which must be based on the natural history of the disease.

Although the presentation of the reasons for choosing the specific type of decision model used is recommended, none of the authors stated their reasons. The description of the models and presentation of the figure or analytical scheme is in Fig. 2.

Results of applying the CHEERS ^18^ to each study are found in the supplementary material (Chart 3).

As for the performance of the screening tests based on accuracy measures, in all economic evaluations, both cervical cytology and HPV DNA tests (rapid test and hybrid capture) showed good specificity. Regarding sensitivity, there was the difference between the HPV-DNA tests and cervical cytology and within cervical cytology (ranging from 58.4%–72%) throughout the studies (supplementary material, Chart 4).

Table 3 shows the interpretation of the results of economic assessments by the classification of the JBI Dominance Matrix, except for two studies: the evaluation carried out by Nahvijou et al.^32^ because it was a cost-minimization study and the study of Levin et al.,^30^ as it did not present data for the analysis.

**Table 3:** Cost-effectiveness of the included studies, according to the Joanna Briggs Institute (JBI) dominance classification matrix.

(+) The intervention is more cost-effective or more effective than the comparator; (0) The intervention has a cost or outcome/benefit equal to the comparator; (-) The intervention is less costly or less effective than the comparator. (D; G; H), Intervention dominance (favorable); (A, E; I), Weak dominance of the intervention; (B; C; F), Non-dominance of the intervention (unfavorable).

The dominance interpretation varied between the studies analyzed (n = 13) or within the same study, depending on the strategies compared.^19^ In 6 studies,^23^^,^^25^, ^26^, ^27^^,^^33^^,^^35^ the HPV test was dominant compared to conventional cytology, which means there would be a favorable decision to incorporate the new test because it represents a lower cost, bringing savings to the health system and with an increase in effectiveness.

Another five studies^22^^,^^28^^,^^29^^,^^35^^,^^36^ showed a weak dominance of the HPV test against cervical cytology, showing greater effectiveness but with a higher cost. In this case, more information is needed on the priorities and preferences of decision-makers, such as ICER values and country cost-effectiveness thresholds.

In particular, the study by Nahvijou et al.^33^ showed that the HPV test had strong or weak dominance, respectively, compared to conventional cytology, depending on the effectiveness measure used, QALY or YLS.

Finally, two studies ^24^^,^^31^ showed that the HPV test was not dominant over conventional cytology and was unfavorable to its incorporation, as it did not present a difference in clinical effectiveness and showed a higher cost. It is noteworthy that in the study conducted by Mandelblatt et al.,^31^ for one of the strategies evaluated (screening every ten years in women starting at 35 years of age and up to 55 years of age), the effectiveness of the HPV test was lower.

## Discussion

This SR analyzed economic assessment studies used to examine the value and performance of testing for CC screening in women from low- and middle-income countries. Of the 15 studies included, the majority were conducted in upper-middle-income countries (71%), underscoring the need for local modeling studies in low- and lower-middle-income countries.

Most of the total economic evaluations were cost-effectiveness analyses (13 studies), in line with another review study.^38^ However, the model selection, the analysis perspective, and the comparative screening strategies varied between the studies and revealed a specific methodological heterogeneity.

The societal perspective is generally recommended because is the most comprehensive and includes costs for the health system, costs for the patient, costs from other sectors, and indirect costs due to loss of productivity. It also allows a complete analysis of all of the opportunity costs attributable to disease and could be preferred for cost analyses such as Cost Benefit Analysis (CBA), Cost Effectiveness Analysis (CEA), and Cost Utility Analysis (CUA). However, societal perspective requires presumably the biggest sizable data, often making it difficult to use in specific contexts.^39^

In this SR all studies adopted the perspective of the health system and the patient, possibly because it is difficult and time-consuming to estimate all cost components from the societal perspective, and because these two perspectives are the most used in economic evaluation studies, precisely because they present a more pragmatic character in answer to a question.^40^

There was a variation in the types of costs used in the different studies. In^10^^,^^22^^,^^24^^,^^24^^,^^27^^,^^29^^,^^31^, ^32^, ^33^, ^34^, ^35^, ^36^ the authors presented only direct medical costs, while in the others, direct medical and non-medical costs were presented.^26^^,^^28^^,^^30^^,^^31^ However, it is important to highlight that the estimate of non-medical direct costs is relevant, especially regarding the costs of patients and families (cost of transport to and from the health service; food, and accommodation, among others).^39^

Indirect costs should also be measured whenever possible, as they involve costs arising from absenteeism, that is, the period the patient is absent from work to receive treatment, or due to lower productivity caused by the effect of the disease or its treatment.^39^

Of the 15 studies included in the SR, 7 (46.67%) used the Markov modeling. Markov models are advantageous in diseases with repeated events over time, such as cancer. Its cyclical nature is convenient to characterize interventions repeated on a scheduled basis over time, as in the CC tracking strategies.^41^ The model will simulate disease progression for a specific cohort of patients, assigning a probability of progression and regression between phases/classifications from dysplasia to invasive cancer.

Five studies (33.34%) that used Decision Tree, the simplest form of analytical models, were included in the SR. In this model, graphic resources are used to describe the possible paths taken by patients if they were under tracking strategies, interventions, or the treatments investigated. These paths include events and their respective probability of occurrence, and in the end, health costs and outcomes are assigned to each path taken.^42^

The limited structure of the Decision Tree model makes its use suitable for an acute disease of a short-time period, however, it reveals difficulties in modeling situations with recurrence of events and long-term periods, as in the case of chronic diseases like CC. In these situations, the use of the Markov Model is recommended.^43^ Studies that used discrete individual-based models, microsimulation, or Semi-Markov models (n = 3; 20%) were also included in this SR.

As for the model parameters, although the performance of the screening tests showed high specificity ranging between 86% and 98%, there was a significant difference in sensitivity between molecular tests based on HPV DNA (Rapid test: 81% to 90%; Hybrid capture: 88% to 95%) and conventional cervical cytology by Pap smear test (range from 58.4% to 72%). These data corroborate the findings of the study of Ginsberg et al.^44^ They show the possibility of distinguishing between the sensitivity and specificity of intra- and inter-regional screening interventions due to differences in method, collection, and professional experience of physicians and laboratory technicians.

It is important for health technology managers and evaluators to be perceptive in adopting measures that prioritize the screening of populations at risk to reduce the cost per year of life saved or the incremental cost-effectiveness ratio.

Health technology assessment studies are permeated by uncertainties related to the model's structure, parameters, and methodology. Most of the studies included only addressed parameter uncertainty by univariate deterministic sensitivity analysis. However, it is necessary to evaluate the effects of model uncertainty on the results.

Screening strategies varied concerning screening age (35, 40, 45 years) and frequency (1, 2, 3 times over a lifetime). The heterogeneity of the intervention and the evaluation outcome measure made it challenging to summarize qualitatively and, above all, quantitatively. In this regard, some authors consider it imprudent to perform a meta-analysis of economic analysis studies, as they believe that producing a robust scientific result is unlikely. This argument is based on variation in the use and costs of resources in different regional scenarios, in the peculiarity of the context (institutional, populational, behavioral, and cultural), and in the multiplicity of methods between the studies, which can interfere with cost-effectiveness measures due to heterogeneity.

The CHEERS instrument,^18^ created to encourage greater standardization and promote better quality in the reports of economic evaluation studies in the health area, guides researchers in preparing the report and includes items in the checklist that ensure greater methodological transparency. However, it is not a tool assessment of the methodological quality itself.

One of the main features of CHEERS ^18^ is that it makes a clear distinction between economic evaluation studies based on a single source of primary data and studies based on modeling for decision analysis with inputs from multiple sources.

In mathematical modeling, the description of the model and its parameters must enable the results to be reproduced by other authors. This is especially relevant when using complex models such as discrete event simulations or Markov microsimulations.

In this SR, regarding the quality of the report, the authors have found that aspects such as the perspective of the study, the comparator; costs and outcomes; findings, and limitations were described in all studies. However, the model's description was considered complete in less than half of the studies, and none of them showed the reasons for their choice.

The absence of reporting the calibration method is possibly due to scarce standards in the calibration of models for cervical cancer screening, lack of consensus in the literature on the minimum specification that should be reported, and the insufficiency of local data to estimate the tracking parameters.^45^^,^^46^

According to Silva et al.,^47^ critical analysis through a script only signals the strengths and weaknesses of a study, and it is up to the evaluator to weigh the results according to the context of each investigation. Thus, it is common to find studies that do not meet all the requirements. However, not considering an item may be a consequence of the lack of available information, but the importance of justification for each point not included in the checklist is highlighted.

To determine the cost-effectiveness of screening, McMeekin et al.^48^ have pointed out that it is necessary to define the optimal age to start screening, if abnormal Pap smears can be better stratified according to risk, and the positive predictive value of the current tracking strategies, among others. The screening interval (every 5, 10 years), coverage (50%‒80%), and adherence or compliance with visits (1, 2, 3 times over a lifetime) have been other aspects evaluated among the compared interventions.

The DRM shows the distribution of studies into three distinct bands, where a predominance of the number of studies in a given band will indicate the likely implication of the intervention. If more studies are located on a matrix space, this would mean a level of dominance associated with that range. However, if there are equal studies in two or three bands, no clear conclusions can be drawn. In this SR, the authors have found that most studies (11/13) are located in the favorable band for the intervention, and the distribution of the number of studies is similar in the classification of strong dominance (n = 6) and weak dominance (n = 5).

This is the main methodological difference between the present study and the SR by Mezei et al.^10^ The authors chose to evaluate the cost-effectiveness results by DRM ^19^ whilst Mezei et al.^10^ only transformed the ICER results of the economic evaluations into international dollars and compared them directly.

Joanna Briggs Institute's analysis of the dominance of strategies by DRM (JBI, 2014) ^19^ in 13 of the studies included allowed us to observe how the results of analyzing benefits and costs between the investigated interventions can vary in different country contexts, with varying results in the same country and even inside the study itself, depending on the strategy. The studies in which the HPV test was dominant compared to conventional cytology^3^^,^^5^^,^^23^^,^^25^^,^^26^^,^^27^^,^^33^ coincided with the authors’ conclusions; however, the elaboration of the dominance matrix allowed us to observe that in the studies conducted by Campos, 2015 and 2017,^25^^,^^26^ the gain in effectiveness measured in years of life saved was tiny (0.005‒0.073 YLS; 0.004‒0.065 YLS; respectively). On the other hand, studies that showed a weak dominance of HPV testing over smear cytology that evaluated greater effectiveness but higher cost ^22^^,^^28^^,^^29^^,^^34^^,^^36^ suggest that, although HPV-DNA testing prices are still very high, they could be negotiated due to the volume of purchase, considering that the HPV-DNA tests were associated with greater efficacy than conventional cytology due to their greater sensitivity and reproducibility. In all cases, the value of the ICER was lower than the cost-effectiveness threshold used (GDP per capita of the countries); however, in at least 2^35^^,^^36^ of them, the effectiveness gain measured by the CIN2+ detected per case or by QALY gained, respectively, was also small (0.001; 0.05), which could suggest a thorough evaluation for decision making in favor of incorporating the new test.

One study ^33^ showed that the HPV-DNA test had strong dominance or weak dominance, respectively, compared to conventional cytology, depending on the measure of effectiveness used, QALY or YLS, pointing in the second case to lower effectiveness despite the lower cost of the HPV-DNA test. This difference may be related to the parameter values used in the model, a limitation mentioned by the study's authors.

The variation in the result presentation is another prominent feature in the studies. Some authors^19^ argue that SR with summarized results from different contexts cannot be extracted, as opportunity costs, resources, comparators, and relevant interventions are very discrepant. However, the SR of economic assessments can be an additional tool for decision-makers, especially in understanding resource allocation and the potential impacts. This can be achieved by identifying gaps in the evidence base, alerts to essential outcomes for intervention selection/compensation, and a better understanding of the circumstances that provide cost-effective models/interventions.^19^

Donaldson et al.^49^ suggest that the SR value of economic analyses is not to generate a single result or reliable recommendation about cost-effectiveness but rather to help decision-makers understand the structure of the resource allocation problem and the potential impacts. Thus, the focus of this article was not to try to generate a summary estimate of the cost-effectiveness relationship but to demonstrate the variability and its determinants from one environment to another.

## Conclusions

The main findings of this review indicate that the HPV-DNA test proved to be cost-effective compared to conventional smear cytology (Pap) in women from low- and middle-income countries for different strategies. Beginning the screening when women are 35 years old, repeating it every five years, and carrying out the test 2 and 3 times throughout life are successful strategies. However, as already discussed, the level of evidence (JBI) of the set of studies showed some disparities related to the types of outcomes evaluated and the types of costs used in the parameters of the models, according to the perspective adopted, which involved in some studies both the perspective of the health system and the patient.

While recognizing that differences in assessment contexts and populations imply that SRs of economic analyses are unlikely to produce unique answers, policymakers, healthcare professionals, patients, and other decision-makers can provide relevant information to choose or trade off the intervention analyzed.

This review is relevant to the public health policy in low- and middle-income countries because it shows evidence that, for most of the studies reviewed, the authors found at least one screening strategy that reduced the incidence of CC at a cost per year of life saved below the per capita GDP of the country investigated, showing the economic feasibility of saving thousands of lives per year by implementing cost-effective tracking strategies.

In terms of research, new screening methods have been proposed, combining CC prevention strategies that include screening and vaccination, bringing methodological challenges to choosing and designing the analytical model in economic evaluations, which are increasingly being used in incorporating technologies. On the other hand, the variability in test accuracy values, specifically from conventional cytology, suggests that new economic assessments could benefit from evidence syntheses or systematic reviews that address this aspect.

The present study is relevant due to the high disease burden of this type of cancer and the number of preventable deaths in women from low- and middle-income countries when there is timely identification of HPV infection through effective screening and access to appropriate treatment.

## Authors’ contributions

CPRC, RCRA, RBL, AMG, MGF, GND and JKV conceived and designed the study protocol; CPRC, RCRA, and RBL contributed to data collection, data analysis, and interpretation, and drafted the manuscript; AMG, MGF, GND, and JKV contributed to critical revision of the manuscript. All authors approved the final version of the manuscript.

## Conflicts of interest

The authors declare no conflicts of interest.

## Appendix Supplementary materials

Image, application 1
