# Supplementary material for: Cervical cancer screening in low- and middle-income countries: A systematic review of economic evaluation studies
Source: Clinics (Sao Paulo). 2022 Jul 26;77:100080. doi: 10.1016/j.clinsp.2022.100080 (PMC9335392; doi:10.1016/j.clinsp.2022.100080)
Supplement: Supplementary file 1 [file mmc1.docx]

**Supplementary Material**

**Chart 1.** Search strategies and results of references retrieved per database.

| **Database** | **Search strategies** | **Results** |
| --- | --- | --- |
| PubMed | (((DNA Probes, HPV[mh] OR Human Papillomavirus DNA Tests[mh] OR "Human Papilloma Virus DNA Probes"[tiab] OR "Human Papilloma Virus DNA Probes"[tiab] OR "HPV DNA Probes"[tiab] OR Cytological Techniques[mh] OR Cell Biology[mh] OR Cytology[sh] OR Cytology[tiab] OR Cytological Techniques OR Cytologic Test*[tiab] OR Cytologic Test*[tiab] OR Cytologic Technique*[tiab] OR Hybrid Capture[tiab] OR Colposcopy[tiab] OR Cobas 4800 HPV Test[tiab] OR HPV Assay*[tiab] OR hrHPV HC2[tiab]) AND (Papanicolaou Test[mh] OR Papanicolaou[tiab] OR Pap Test[tiab] OR Pap Smear[tiab])) AND (Uterine Colo de útero Neoplasms[mh] OR ((Colo de útero[tiab] OR Uterine Colo de útero[tiab] OR Cervix[tiab]) AND (Neoplas*[tiab] OR Cancer*[tiab] OR Carcinoma*[tiab] OR Adenocarcinoma*[tiab] OR Tumor*[tiab] OR Tumour*[tiab] OR Malignan*[tiab]))) AND (Mass Screening[mh] OR Diagnosis[mh] OR Screening[tiab] OR Diagnos*[tiab])) AND (Economics[majr:noexp] OR “costs and cost analysis”[majr] OR (economic[tiab] AND model*[tiab]) OR cost minimi*[tiab] OR cost-utilit*[tiab] OR health utilit*[tiab] OR economic evaluation*[tiab] OR economic review*[tiab] OR cost outcome[tiab] OR cost analys*[tiab] OR economic analys*[tiab] OR (budget*[tiab] AND impact analys*[tiab]) OR cost-effective*[ti] OR pharmacoeconomic*[ti] OR pharmaco-economic*[ti] OR cost-benefit[ti] OR costs[ti] OR cost-effective*[ot] OR pharmacoeconomic*[ot] OR pharmaco-economic*[ot] OR cost-benefit[ot] OR costs[ot] OR life year[tiab] OR life years[tiab] OR qaly*[tiab] OR cost-benefit analys*[tiab] OR cost-effectiveness analys*[tiab] OR ((cost[ti] OR economic*[ti] OR cost[ot] OR economic*[ot]) AND (costs[tiab] OR cost-effectiveness[tiab] OR markov[tiab]))) NOT Review[tw] | 174 |
| Embase | ('papanicolaou test'/exp OR 'pap smear':ti,ab OR 'papanicolaou test':ti,ab OR 'pap stain':ti,ab OR 'pap test':ti,ab OR 'papanicolaou cytology':ti,ab OR 'papanicolaou method':ti,ab OR 'papanicolaou smear':ti,ab OR 'papanicolaou stain':ti,ab) AND ('human papillomavirus dna test'/exp OR 'amplicor hpv test':ti,ab OR 'amplicor human papillomavirus test':ti,ab OR 'anyplex ii':ti,ab OR 'cintec plus kit':ti,ab OR 'cervista':ti,ab OR 'clart hpv 2':ti,ab OR 'clart hpv line':ti,ab OR 'cobas 4800 hpv test':ti,ab OR 'cobas hpv test':ti,ab OR 'hpv dna tests':ti,ab OR 'human papillomavirus dna test':ti,ab OR 'human papillomavirus dna test kit':ti,ab OR 'human papillomavirus dna tests':ti,ab OR 'hybrid capture 1':ti,ab OR 'hybrid capture 2':ti,ab OR 'inno lipa hpv genotyping extra':ti,ab OR 'linear array hpv genotyping test':ti,ab OR 'linear array (device)':ti,ab OR 'papillocheck':ti,ab OR 'pretect':ti,ab OR 'pretect hpv-proofer':ti,ab OR 'realtime hpv assay':ti,ab OR 'realtime high-risk hpv':ti,ab OR 'carehpv':ti,ab OR 'cytology'/exp OR 'automated cytological technique':ti,ab OR 'cell biology':ti,ab OR 'cytological techniques':ti,ab OR 'cytology':ti,ab OR 'cytotechnology':ti,ab OR 'cytotest':ti,ab OR 'cytological test*':ti,ab OR 'molecular test*':ti,ab OR 'hpv test*':ti,ab) AND ('uterine cervix cancer'/exp OR 'cancer, uterine cervix':ti,ab OR 'colo de útero cancer':ti,ab OR 'cervix ca':ti,ab OR 'cervix cancer, recurrent':ti,ab OR 'cervix cancer, uterine':ti,ab OR 'cervix malignancy, recurrent':ti,ab OR 'cervix uteri cancer':ti,ab OR 'cervix uterus cancer':ti,ab OR 'neoplasma cervicis recurrens':ti,ab OR 'neoplasma cervicis uteri recurrens':ti,ab OR 'recurrent cervix cancer':ti,ab OR 'recurrent cervix malignancy':ti,ab OR 'uterine colo de útero cancer':ti,ab OR 'uterine cervix cancer':ti,ab OR 'uterine cervix cancer, recurrent':ti,ab OR 'uterine cervix malignancy, recurrent':ti,ab OR 'uterus cervix cancer':ti,ab OR 'uterine cervix tumor'/exp OR 'cervix neoplasia':ti,ab OR 'cervix neoplasms':ti,ab OR 'cervix tumor':ti,ab OR 'cervix tumour':ti,ab OR 'uterine colo de útero neoplasms':ti,ab OR 'uterine cervix neoplasm':ti,ab OR 'uterine cervix tumor':ti,ab OR 'uterine cervix tumour':ti,ab OR 'cervix cancer':ti,ab OR 'uterine cervix carcinoma'/exp OR 'advanced uterine cervix carcinoma':ti,ab OR 'carcinoma cervicis uteri':ti,ab OR 'carcinoma cervicis uteri recurrens':ti,ab OR 'carcinoma colli uteri':ti,ab OR 'colo de útero carcinoma':ti,ab OR 'cervix carcinoma':ti,ab OR 'cervix carcinoma recidivism':ti,ab OR 'cervix carcinoma stage 1 a':ti,ab OR 'cervix carcinoma, inoperable':ti,ab OR 'cervix carcinoma, recurrent':ti,ab OR 'cervix carcinoma, uterine':ti,ab OR 'cervix squamous cell carcinoma':ti,ab OR 'cervix uteri carcinoma':ti,ab OR 'cervix uteri carcinoma, recurrent':ti,ab OR 'endocolo de útero carcinoma':ti,ab OR 'endocervix carcinoma':ti,ab OR 'recidivism, uterine cervix carcinoma':ti,ab OR 'recurrent carcinoma cervix uteri':ti,ab OR 'recurrent carcinoma uterine cervix':ti,ab OR 'recurrent carcinoma, cervicis uteri':ti,ab OR 'recurrent cervix carcinoma':ti,ab OR 'recurrent uterine cervix carcinoma':ti,ab OR 'uteri cervix carcinoma':ti,ab OR 'uterine colo de útero carcinoma':ti,ab OR 'uterine cervix carcinoma':ti,ab OR 'uterine cervix carcinoma recidivism':ti,ab OR 'uterine cervix carcinoma stage i':ti,ab OR 'uterine cervix carcinoma state':ti,ab OR 'uterine cervix invasive carcinoma':ti,ab OR 'uterine cervix squamous cell carcinoma':ti,ab OR 'uterine cervix stump carcinoma':ti,ab OR 'uterine endocervix carcinoma':ti,ab OR 'uterus cervi carcinoma':ti,ab OR 'uterus cervix carcinoma':ti,ab OR 'cervix neoplasm*':ti,ab) AND ('screening'/exp OR 'multiple screening':ti,ab OR 'prescreening':ti,ab OR 'project, screening':ti,ab OR 'screening':ti,ab OR 'screening method':ti,ab OR 'screening procedure':ti,ab OR 'screening program':ti,ab OR 'screening programme':ti,ab OR 'screening project':ti,ab OR 'mass screening'/exp OR 'health screening':ti,ab OR 'health screening program':ti,ab OR 'health screening programme':ti,ab OR 'longitudinal health screening program':ti,ab OR 'longitudinal health screening programme':ti,ab OR 'mass screening':ti,ab OR 'population screening':ti,ab OR 'screening, mass':ti,ab) AND ('economic evaluation'/exp OR 'economic evaluation':ti,ab OR 'economics'/exp OR 'economics':ti,ab OR 'economic stud*':ti,ab OR 'cost effectiveness analysis'/exp OR 'cost effectiveness':ti,ab OR 'cost effectiveness analysis':ti,ab OR 'cost effectiveness ratio':ti,ab OR 'cost efficiency analysis':ti,ab OR 'cost benefit analysis'/exp OR 'cost analysis':ti,ab OR 'cost benefit':ti,ab OR 'cost benefit analysis':ti,ab OR 'cost benefit ratio':ti,ab OR 'cost-benefit analysis':ti,ab OR 'markov chain'/exp OR 'markov chain':ti,ab OR 'markov chains':ti,ab OR 'markov model':ti,ab OR 'markov process':ti,ab OR 'markov chain monte carlo method'/exp OR 'markov chain monte carlo':ti,ab OR 'markov chain monte carlo method':ti,ab OR 'markov':ti,ab OR 'pharmacoeconomics'/exp OR 'economics, pharmaceutical':ti,ab OR 'pharmaceutical economics':ti,ab OR 'pharmaco-economic analysis':ti,ab OR 'pharmaco-economic evaluation':ti,ab OR 'pharmaco-economics':ti,ab OR 'pharmacoeconomic analysis':ti,ab OR 'pharmacoeconomic evaluation':ti,ab OR 'pharmacoeconomics':ti,ab) AND [embase]/lim NOT ([embase]/lim AND [medline]/lim) | 168 |
| CRD | ((DNA Probes, HPV OR Human Papillomavirus DNA Tests OR Human Papilloma Virus DNA Probes OR HPV DNA Probes OR Cytological Techniques OR Cell Biology OR Cytology OR Cytologic Tests OR Hybrid Capture OR Colposcopy OR Cobas 4800 HPV Test OR HPV Assays OR hrHPV HC2) AND (Papanicolaou Test OR Papanicolaou OR Pap Test OR Pap Smear) AND (Colo de útero OR Uterine Colo de útero OR Cervix)) and ((Economic evaluation:ZDT and Bibliographic:ZPS) OR (Economic evaluation:ZDT and Abstract:ZPS) OR Project record:ZDT OR Full publication record:ZDT) IN NHS EED, HTA | 70 |
| BVS Regional/ Lilacs | (tw:("Papanicolaou Test" OR Papanicolaou OR "Pap Test" OR "Pap Smear" OR Papanicolau)) AND (tw:("DNA Probes, HPV" OR "Human Papillomavirus DNA Tests" OR "Human Papilloma Virus DNA Probes" OR "HPV DNA Probes" OR "Cell Biology" OR Cytology OR Cytologic OR Cytological OR "Hybrid Capture" OR Colposcopy OR "Cobas 4800 HPV Test" OR "HPV Assay" OR "hrHPV HC2" OR Citologia OR "Captura Híbrida" OR Colposcopia OR "Cobas 4800 HPV Teste" OR "HPV Tests" OR "HPV Test")) AND (tw:("Mass Screening" OR Diagnos* OR Screening OR "Deteccao de massa" OR diagnóstico OR deteccao OR "Detección masiva" OR diagnóstico* OR detección)) AND (tw:("Health Evaluation" OR "Evaluación en Salud" OR "Avaliacao em Saúde" OR "Avaliacao Econômica em Saúde" OR "Economic Evaluation" OR Economic* OR "Avaliacao Economica" OR "cost analysis" OR "economic analysis" OR "análise de custos" OR "análise econômica" OR "análisis de costos" OR "análisis económico" OR "cost-effective" OR pharmacoeconomic* OR "custo efetividade" OR farmacoeconomia OR "cost-effectiveness" OR "cost-effectiveness analys" OR "cost benefit" OR "Evaluación costo-efectividad" OR "impacto económico" OR Custo OR Costo OR Cost OR Markov)) AND (db:("LILACS")) | 55 |
| **Total** |  | 467 |

**Chart 2.** Characteristics of excluded studies.

| Studies | Reasons for exclusion |
| --- | --- |
| Aarnio 2020 | High-income country population |
| Bleggi Torres 2003 | Assessing the costs and consequences of a tracking program |
| Campos 2015 | The intervention was visual inspection with acetic acid followed by treatment with cryotherapy for all eligible women (regardless of outcome); Pap smear was not performed in Phase I of the study |
| Cantor 1998 | Comparison of diagnostic strategies (not include molecular HPV testing |
| Chanthavilay 2016 | Evaluation of screening strategies combined with a vaccination program to cervical cancer prevention for 10-year-old girls |
| Chen 2011 | High-income country population |
| Chesebro 1996 | High-income country population |
| Chuck 2010 | High-income country population |
| Corona 2016 | Congress abstract, no complete data |
| Endarti 2017  (pharmacoeconomic) | Congress abstract, no complete data |
| Endarti 2017  (Markov model) | Congress abstract, no complete data |
| Gamzu 2002 | High-income country population |
| Ginsberg 2009 | Screening program evaluation |
| Guerrero 2015 | Interventions evaluated were vaccination and screening performed by visual inspection of the cervix with acetic acid |
| Hernández-Peña 1997 | Screening program evaluation |
| Hsaïri 2000 | Study not found in full |
| Hughes 2005 | High-income country population |
| Kececioglu 2013 | High-income country population |
| Kim 2008 | The intervention was vaccination combined with three screening test availability scenarios in both regions (North and South Vietnam) |
| Kim 2017 | High-income country population |
| Kulasingam 2006 | High-income country population. |
| Lairson 2014 | Incremental cost assessment per woman who underwent Pap smear |
| Levin 2002 | Commentary article |
| Lince-Deroche 2015 | HIV+ female population |
| Lonky 2007 | High-income country population |
| Lynch 2004 | High-income country population |
| Lytwyn 2004 | High-income country population |
| Malenie 2020 | Congress abstract, no complete data |
| Mandelblatt 1988 | High-income country population |
| Mann 2014 | HIV+ female population, congress abstract without complete data |
| Mann 2015 | HIV+ female population, congress abstract without complete data |
| Maxwell 2002 | High-income country population |
| Myers 2000 | High-income country population |
| Nokiani 2008 | Cross-sectional study |
| Oliver 2002 | Study not found in full |
| Perovic 2009 | Study not found in full |
| Praditsitthikorn 2011 | The interventions evaluated were vaccination and screening performed by visual inspection of the cervix with acetic acid |
| Sato 1999 | No HPV DNA test performed |
| Schweitzer 1974 | No HPV DNA test performed |
| Scoggins 2010 | No HPV DNA test performed |
| Sikon 2014 | Congress abstract, no complete data |
| Sharma 2011 | Evaluating study about the cost-effectiveness of HPV vaccination in pre-adolescents and screening between one and five times in adulthood; and the cost-effectiveness of combined vaccination and screening |
| Straughn 2004 | Cost study |
| Suba 2001 | Comparison between screening (Pap smear) and no screening |
| Taylor 2000 | No HPV DNA test performed |
| Teixeira 2020 | Protocol |
| van Ballegooijen 1997 | Cost study |
| Vijayaraghavan 2009 | 50% of the female population as HIV+ |
| Vodicka 2017 | Cost study |
| Vooijs 1998 | Editorial |
| Woo 2007 | The study evaluated the optimization (3, 4, or 5 years) of screening for cervical cancer through Pap smear, does not evaluate HPV test |
| Xu 2013 | Study not found in full |
| Zeferino 2018 | Congress abstract, no complete data |
| Zhao 2012 | Study not found in full |
| Zimmermann 2017 | HIV+ female population |

**Chart 3.** Items rated, according to CHEERS.

| **Section/ item** | **Andrés-Gamboa (2008)** | **Beal  (2014)** | **Caetano  (2006)** | **Campos  (2015)** | **Campos  (2017)** | **Flores  (2011)** | **Goldie  (2005)** | **Gutiérrez-Delgado (2008)** | **Levin  (2010)** | **Mandelblatt  (2002)** | **Nahvijou  (2014)** | **Nahvijou  (2016)** | **Tantitamit  (2015)** | **Termrungruanglert (2017)** | **Termrungruanglert (2019)** |
| --- | --- | --- | --- | --- | --- | --- | --- | --- | --- | --- | --- | --- | --- | --- | --- |
| **Title** | YES | PARTIAL | YES | PARTIAL | YES | YES | YES | YES | PARTIAL | YES | YES | YES | PARTIAL | YES | YES |
| **Abstract** | PARTIAL | PARTIAL | PARTIAL | YES | PARTIAL | PARTIAL | PARTIAL | YES | YES | YES | PARTIAL | PARTIAL | PARTIAL | YES | YES |
| **Background and  objectives** | YES | PARTIAL | YES | YES | PARTIAL | YES | PARTIAL | YES | YES | YES | YES | YES | YES | YES | YES |
| **Target population and subgroups** | YES | YES | YES | YES | YES | YES | YES | YES | YES | YES | YES | YES | PARTIAL | YES | YES |
| **Setting and location** | YES | YES | YES | NO | YES | YES | NO | YES | YES | NO | NO | NO | YES | YES | YES |
| **Study perspective** | YES | YES | YES | YES | YES | YES | YES | YES | YES | YES | YES | YES | YES | YES | YES |
| **Comparators** | YES | YES | YES | YES | YES | YES | YES | YES | YES | YES | YES | YES | YES | YES | YES |
| **Time horizon** | YES | YES | YES | YES | YES | YES | YES | YES | YES | PARTIAL | NO | YES | NO | YES | YES |
| **Discount rate** | YES | YES | YES | YES | YES | YES | YES | YES | YES | YES | NO | YES | NO | YES | YES |
| **Choice of health outcomes** | YES | YES | NO | YES | YES | YES | YES | YES | PARTIAL | YES | NO | YES | NO | YES | YES |
| **Measurement of effectiveness** | YES | YES | YES | YES | YES | YES | YES | YES | YES | YES | YES | YES | NO | YES | YES |
| **Measurement and valuation of preference based outcomes** | NO | NO | NO | NO | NO | NO | NO | NO | NO | NO | NO | NO | NO | NO | NO |
| **Estimating resources and costs** | YES | YES | YES | YES | YES | YES | YES | YES | YES | YES | YES | YES | NO | YES | YES |
| **Currency, price date, and conversion** | YES | YES | YES | YES | YES | YES | YES | PARTIAL | YES | PARTIAL | NO | PARTIAL | NO | YES | YES |
| **Choice of model** | PARTIAL | YES | PARTIAL | PARTIAL | YES | YES | YES | YES | YES | PARTIAL | YES | PARTIAL | PARTIAL | PARTIAL | YES |
| **Assumptions** | YES | YES | YES | YES | YES | YES | YES | YES | YES | YES | NO | YES | NO | YES | YES |
| **Analytical methods** | NO | NO | NO | PARTIAL | NO | NO | NO | NO | YES | NO | NO | NO | NO | NO | NO |
| **Study parameters** | YES | YES | YES | YES | YES | YES | NO | YES | YES | YES | PARTIAL | YES | NO | YES | YES |
| **Incremental costs and outcomes** | YES | YES | YES | YES | YES | YES | YES | YES | YES | YES | YES | YES | YES | YES | YES |
| **Characterizing uncertainty** | YES | YES | YES | YES | YES | YES | YES | YES | YES | YES | NO | YES | NO | YES | YES |
| **Characterising heterogeneity** | NO | NO | NO | YES | NO | NO | NO | NO | YES | NO | NO | NO | YES | YES | YES |
| **Study findings, limitations, generalisability, and current knowledge** | YES | YES | YES | YES | YES | YES | YES | YES | YES | YES | YES | YES | YES | YES | YES |
| **Source of funding** | NO | NO | NO | YES | NO | YES | PARTIAL | NO | YES | YES | PARTIAL | PARTIAL | NO | YES | YES |
| **Conflicts of interest** | NO | YES | NO | YES | YES | NO | NO | NO | YES | NO | NO | NO | NO | YES | YES |

**Chart 4. Performance of screening tests used in the studies.**

| **Accuracy** | **Sensitivity** | **Specificity** |
| --- | --- | --- |
| Cervical cytology | 58.4 – 72% | 86 – 98% |
| HPV-DNA rapid test | 81 – 90% | 86 – 95% |
| HPV DNA hybrid capture | 88 – 95% | 86 – 95% |
